# Time-Dependent Distribution of Hydroxychloroquine in Cynomolgus Macaques Using Population Pharmacokinetic Modeling Method

**DOI:** 10.3389/fphar.2020.602880

**Published:** 2021-01-14

**Authors:** Qi Liu, Guofang Bi, Guiying Chen, Xuan Guo, Siqi Tu, Xiaolin Tong, Man Xu, Mengjie Liu, Bei Wang, Hongliang Jiang, Jufeng Wang, Haiyan Li, Kun Wang, Dongyang Liu, Chunli Song

**Affiliations:** ^1^ Department of Orthopedics, Peking University Third Hospital, Beijing, China; ^2^ Drug Clinical Trial Center, Peking University Third Hospital, Beijing, China; ^3^ HR-Biopharm Technology Co., Ltd., Wuhan, China; ^4^ Pharmaron Beijing Co., Ltd., Beijing, China; ^5^ Shanghai Qiangshi Information Technology Co., Ltd., Shanghai, China

**Keywords:** hydroxychloroquine, biodistribution, population pharmacokinetics, cynomolgus macaques, time-dependent

## Abstract

To evaluate the biodistribution of hydroxychloroquine (HCQ) in cynomolgus macaques and receive dynamic quantitative relationship between plasma, blood, and lung tissue concentration using the population pharmacokinetic modeling method, seventeen cynomolgus macaques were divided into six groups according to different HCQ dosing regimens over 5 days. The monkeys were euthanized, and blood, plasma, urine, feces and ten tissues were collected. All the samples were prepared by protein precipitation and analyzed by HPLC-MS/MS detection. The population pharmacokinetics of HCQ in the plasma, red blood cells, and lung tissue was conducted and simulated via ADAPT program. Results demonstrated that the maximum concentration (*C*_max_) of HCQ was 292.33 ng/mL in blood and 36.90 ng/mL in plasma after single dose of 3 mg/kg. The value of area under curve (AUC_0–∞_) was determined as 5,978.94 and 363.31 h* ng/mL for the blood and plasma, respectively. The descending order of the tissue-to-plasma concentration ratio was liver > spleen > kidney > lung > heart > subcutaneous fat > brain. The tissue-to-plasma concentration ratio and the tissue-to-blood concentration ratio for lung were found to be time-dependent with 267.38 and 5.55 at 120 h postdose, respectively. A five-compartment model with first-order oral absorption and elimination best described the plasma, blood, and lung tissue pharmacokinetics. The estimated elimination rate constant (ke) for a typical monkey was 0.236 h^−1^. The volume of distribution in central (Vc/F) and other two peripheral compartments (Vb/F and Vl/F) were 114, 2.68, and 5.55 L, respectively. Model-based simulation with PK parameters from cynomolgus macaques showed that the ratio of the blood or plasma to lung tissue was a dynamic change course, which suggested that the rate of HCQ concentration decrease in the blood or plasma was faster than that in the lung tissue. HCQ was found to be accumulated in tissues, especially in the liver, kidney, lung, and spleen. Also, the tissue-to-plasma concentration ratio increased over time. The population pharmacokinetic model developed could allow for the assessment of pharmacokinetics–pharmacodynamics relationships, especially relevant tissue concentration-response for HCQ. Determining appropriate treatment regimens in animals allows translation of these to clinical studies.

## Introduction

As a 4-aminoquinoline compound with the hydroxyl group based on structure of chloroquine, hydroxychloroquine (HCQ) has a similar therapeutic effect but less side effects compared with other antimalarial drugs. Several clinical practices confirmed that HCQ has good safety and reliable efficacy in various rheumatic diseases such as systemic lupus erythematosus and rheumatoid arthritis. Furthermore, it has been used as basic treatment for rheumatic immune diseases.

Since the outbreak of the novel coronavirus disease (COVID-19) in December 2019, which is caused by severe acute respiratory syndrome coronavirus 2 (SARS-CoV-2), there are more than 21.2 million confirmed cases of those infected and nearly 770,000 deaths (WHO Situation Report, 2019). It has been confirmed that HCQ has a relatively strong anti-SARS-CoV-2 effect in several nonclinical studies (Liu et al., 2020; Wang et al., 2020; Yao et al., 2020). In addition, clinical research on the treatment of HCQ has been carried out around the world. However, there are differences in the HCQ dosing regimens among these clinical trials, and the effectiveness and safety of HCQ are still controversial (Gao et al., 2020; Gautret et al., 2020; Mehra et al., 2020; Molina et al., 2020). Therefore, the dosing regimen optimization of HCQ has become a key point in its clinical application. Due to the target site for the COVID-19 treatment mainly focus on the lung tissue and the transport mechanism of HCQ is similar to active transport (Xu et al., 2016), the blood concentration or plasma concentration of HCQ does not represent that in lung tissue. In addition, there are a broad range of EC_50_ values with different cell lines or different sampling times. Thus, it is necessary to conduct additional pharmacokinetic studies to understand how the concentration in the lung tissue relates to its efficacy.

However, there are still some difficulties in clinical studies, such as the ethical issues and operability of obtaining tissue samples. Physiologically based pharmacokinetic (PBPK) modeling, a powerful means of optimizing the dosing regimen, can integrate the drug physicochemical properties, *in vitro* experimental data, and human physiological parameters to predict the pharmacokinetic characteristics of the drug *in vivo* in a mechanistic manner.

In this study, we first established a high-performance liquid chromatography tandem mass spectrometry (HPLC-MS/MS) method for determining the concentration of HCQ and N-desethyl hydroxychloroquine (DHCQ) in cynomolgus monkeys. Then, we performed a biodistribution study following different HCQ dosing regimens in monkeys. We also conducted a population pharmacokinetics study to characterize the time-dependent pharmacokinetic characteristics, and then HCQ concentration in the lung, blood, and plasma were predicted so as to support the verification of HCQ physiologically based pharmacokinetic (PBPK) model to assist clinical dosing regimen optimization.

## Materials and Methods

### Materials and Reagents

The reference standard of hydroxychloroquine sulfate (100% purity), hydroxychloroquine sulfate-D4 (internal standard, IS), and N-desethyl hydroxychloroquine (DHCQ) bromide were purchased from TLC Pharmaceutical Standards Ltd. (Newmarket, Ontario, Canada). Hydroxychloroquine sulfate (99.90% purity), the experimental drug, was supplied by Jiangsu Shenhua Pharmaceutical Co., Ltd. Ammonium formate of analytical grade was purchased from Sinopharm Chemical Reagent Co., Ltd. (Beijing, China). Methanol, acetonitrile, and formic acid of HPLC grade were purchased from Fisher Scientific (Shanghai, China). Purified water obtained from a PURELAB Option-Q7 (ELGA LabWater, High Wycombe, United Kingdom) system was used throughout the experiment.

### Experimental Animals

Cynomolgus macaques (4.13 ± 0.43 kg) were supplied by Shanghai Beijing Institute of Xieerxin Biology Resource (SCXK: 2015-0011). The animal experiments were carried out in accordance with the guidance from the Animal Care and Use Committee of the Pharmaron Beijing Co. Ltd. (IACUC No.16-120).

### Equipment and HPLC-MS/MS Conditions

Biological samples were analyzed using an HPLC–MS/MS system. The liquid chromatography system (Shimadzu, Japan) was equipped with an LC-20ADXR binary liquid pump. Chromatographic separation was performed on a Venusil C18 Plus chromatographic column (5 µm, 2.1 × 50 mm, Agela), and the column temperature was maintained at 35 °C. The mobile phase consisted of 0.1% formic acid and 20 mM ammonium formate in water (mobile phase A) and acetonitrile (mobile phase B). The chromatographic separation was achieved using 3-min gradient elution. The gradient was 5% B at 0.0–0.01 min, 5%–30% B at 0.01–1.40 min, 30%–95% B at 1.40–1.50 min, 95% B at 1.50–2.30 min, 95%–5% B at 2.30–2.40 min, and 5% B at 2.40-3.00 min. The flow rate was set to 0.8 mL/min. The injection volume was 2.0 µL for all samples.

Mass spectrometric detection was performed on an AB Triple Quad 4500 mass spectrometer, equipped with an electrospray ionization (ESI) source (Turbo Spray ESI source) and Analyst 1.6.3 quantitative analysis software (American Applied Biosystems company). The ESI source was set in the positive mode, with a spray voltage of 5500 V. The ion source temperature was set to 600 °C. The pressure of ion source gas 1 (N_2_) and ion source gas 2 (N_2_) was both set to 50 psi. The air curtain gas (N_2_) pressure was 30 psi. Optimal multiple reaction monitoring (MRM) was selected, and the dwell time for each analyte was 150 ms. The ion pairs used for quantitative analysis were m/z 336.1→247.0 (hydroxychloroquine), m/z 308.2→247.0 (N-desethylhydroxychloroquine), and m/z 340.1→247.0 (IS, hydroxychloroquine-D4). The collision energy (CE) was set to 30 eV for each analyte.

### Sample Preparation

For blood samples, an aliquot of 50 µL whole blood or plasma was mixed with 25 µL IS (500 ng/mL) solution and 50 µL zinc sulfate solution (0.2 mol/L). After thorough vortex mixing for 1 min, 400 µL acetonitrile containing 2% formic acid was added for protein precipitation. After thorough vortex mixing for 1 min, the samples were centrifuged at 1700g for 15 min, an aliquot containing 100 µL of the supernatant was diluted with 200 µL 90% acetonitrile (acetonitrile: H_2_O, 9:1, v/v), and the diluent was collected for HPLC-MS/MS analysis after thorough vortex mixing for 1 min. While for the plasma samples, the preparation method was the same as the blood samples, but without zinc sulfate solution.

Cynomolgus macaque (male, 7–8 weeks old, weight: 4.0 ± 0.8 kg) tissue samples were removed from the -70 °C refrigerator and placed on the ice tray to be cut into pieces. Tissues were placed in the homogenate tubes, and 10 times equivalent volume of 50% methanol (methanol: H_2_O, 1: 1, v/v) was added. The mixture was homogenized with a high-speed homogenizer, and the produced homogenate was stored at -70 °C until analysis. Similar to the preparation of the plasma, the tissue samples (50 µL) were mixed with 25 µL of IS (500 ng/mL) and 400 µL of acetonitrile containing 2% formic acid, then vortex mixed for 1 min, and centrifuged at 14,000 rpm for 5 min. An aliquot of 100 µL of the supernatant was diluted with 200 µL 90% acetonitrile (acetonitrile: H_2_O, 9:1, v/v), and the diluent was then analyzed by LC-MS/MS.

## Method Validation

The selectivity, linearity, accuracy, precision, extraction recovery, and stability of the LC–MS/MS method used in the present study were evaluated as follows. The selectivity of this method was examined by analyzing the MRM chromatograms of blank monkey blood/plasma/tissue homogenate, blank monkey blood/plasma/tissue homogenate spiked with HCQ, DHCQ and IS, and monkey blood/plasma/tissue homogenate collected at indicated time after intragastric administration of hydroxychloroquine sulfate and spiked with IS.

The calibration samples of analytes were prepared by adding a series of different concentrations of working solution and IS working solution to monkey blood, plasma, or tissue homogenate to determine the linearity and LLOQ, then processed as described in “Section 2.4.” The calibration concentrations used for HCQ/DHCQ were 5.00/2.50, 10.00/5.00, 25.00/12.50, 50.00/25.00, 150.00/75.00, 500.00/150.00, 900.00/450.00, and 1,000.00/500.00 ng/mL in blood samples and 2.00/0.20, 4.00/0.40, 10.00/1.00, 20.00/2.00, 50.00/5.00, 100.00/10.00, 180.00/18.00, and 200.00/20.00 ng/mL in plasma samples. Additionally, the calibration concentrations of HCQ and DHCQ in tissue homogenate were 0.40, 0.80, 2.00, 5.00, 20.00, 180.00, and 200.00 ng/mL. Carryover of the tested analytes was assessed by injection of blank plasma samples after injection of the highest level calibration standard. The accuracy and precision assay for intraday and interday were evaluated by the analysis of three QC samples (n = 6) on the same day and on three consecutive days. The precision is expressed by the relative standard deviation (RSD).

The extraction recovery was determined by comparing the peak areas of the QC samples prespiked in blank blood, plasma, and tissue homogenates with those in the mobile phase (n = 6). The matrix effect was evaluated by comparing the peak areas of the QC samples postspiked in the blank blood, plasma, and tissue homogenate with those in the mobile phase (n = 6). The extraction recovery and matrix effect of the IS were determined in the same way. The stability of the tested analytes was evaluated using the QC samples spiked in blank monkey biological samples (n = 3) under the following conditions: (1) store at room temperature for 24 h; (2) three freeze-thaw cycles from −80 °C to room temperature; and (3) store at−80 °C for 30 days.

### Pharmacokinetics Analysis

Cynomolgus macaques (male, weight: 4.13 ± 0.13 kg) were randomly divided into six groups from A to F. In group A, monkeys were given with 3 mg/kg hydroxychloroquine. In group B, monkeys were treated with 3 mg/kg hydroxychloroquine twice at D1 and D2, with an interval of 4 h. In group C, 2 mg/kg hydroxychloroquine was administered twice at D1, with an interval of 4 h, and then 1 mg/kg hydroxychloroquine twice daily was treated at D2 and D3, with an interval of 4 h. In group D, 6 mg/kg hydroxychloroquine was administered twice at D1, with an interval of 4 h, and then 2 mg/kg hydroxychloroquine twice daily was treated at D2 and D3, with an interval of 4 h. In group E, 21 mg/kg hydroxychloroquine was administered twice at D1, with an interval of 4 h. In group F, 21 mg/kg hydroxychloroquine was administered twice at D1, with an interval of 4 h, and then 7 mg/kg hydroxychloroquine twice daily was treated at D2∼D5, with an interval of 4 h. Hydroxychloroquine was administered intragastrically in all groups.

Blood samples (approximately 2 mL) were collected before and after dosing from the hind limb vein in polyethylene tubes at 1, 2, 4, 8, 24, 48, and 72 h with K_2_-EDTA as anticoagulant in groups A ∼ D. About 2 mL blood samples were collected at predose and 1, 2, 4, 8, 24, 48, 72, 144, 192, 240, and 264 h postdose with the same anticoagulant in group E. And about 2 mL blood samples were collected at predose and 1, 2, 4, 8, 24, 48, 72, 96, 97, 98, 100, 104, and 120 h postdose with the same anticoagulant in group F. Then, 2 mL blood sample was divided equally into two parts, one of which was stored at -70 °C until analysis without any treatment, while the other one was centrifuged at 2000 g for 10 min at 4 °C, and then, the harvested plasma samples were stored at -70 °C until analysis. The pharmacokinetic parameters t_1/2_ (the biological half-life), CLz (total body clearance), and AUC_0–t_ (area under curve)were calculated from the plasma concentration–time data according to noncompartmental methods by WinNonlin (Version 8.1, Pharsight, Mountain View, CA) in a noncompartmental manner.

### Tissue Distribution

Cynomolgus macaques (male, weight: 4.13 ± 0.13 kg) were randomly divided into six groups (n = 3 for each group, except group F (n = 2)). All individuals in each group were euthanized at 504, 336, 192, 240, 264, and 120 h following administration of hydroxychloroquine. Tissues (including the lung, bronchoalveolar fluid, liver, kidney, heart, spleen, brain, subcutaneous fat, and thymus) were harvested and homogenized by the methods described in “Section 2.4.” Each monkey was given 50 mL of normal saline irrigation for both lungs. All these samples were then stored at -70 °C until analysis.

### Population PK Model Development

#### Subjects and Study Design

Total 17 cynomolgus macaques were included in this analysis. The dose regimen administered including a) 11.82–11.97 mg (3 individuals with dose time: 0, 4, 24, and 28 h), b) the first two loading doses in the range of 8.12–9.4 mg, following the 4 dose in the range of 4.06–4,72 mg (3 individuals with dose time: 0, 4, 24, 28, 48, and 52 h), c) first two loading doses in range of 24.72–27.78 mg, following the 4 dose in the range of 8.24–9.26 mg (3 individuals with dose times 0, 4, 24, 28, 48, and 52 h). Intensive plasma (141) and blood sampling (149) were performed. Fourteen of 17 cynomolgus macaques with one sample from the lung were collected at 120, 198, 240, 264, and 480 h.

#### Structural PK Model

**FIGURE 1:**
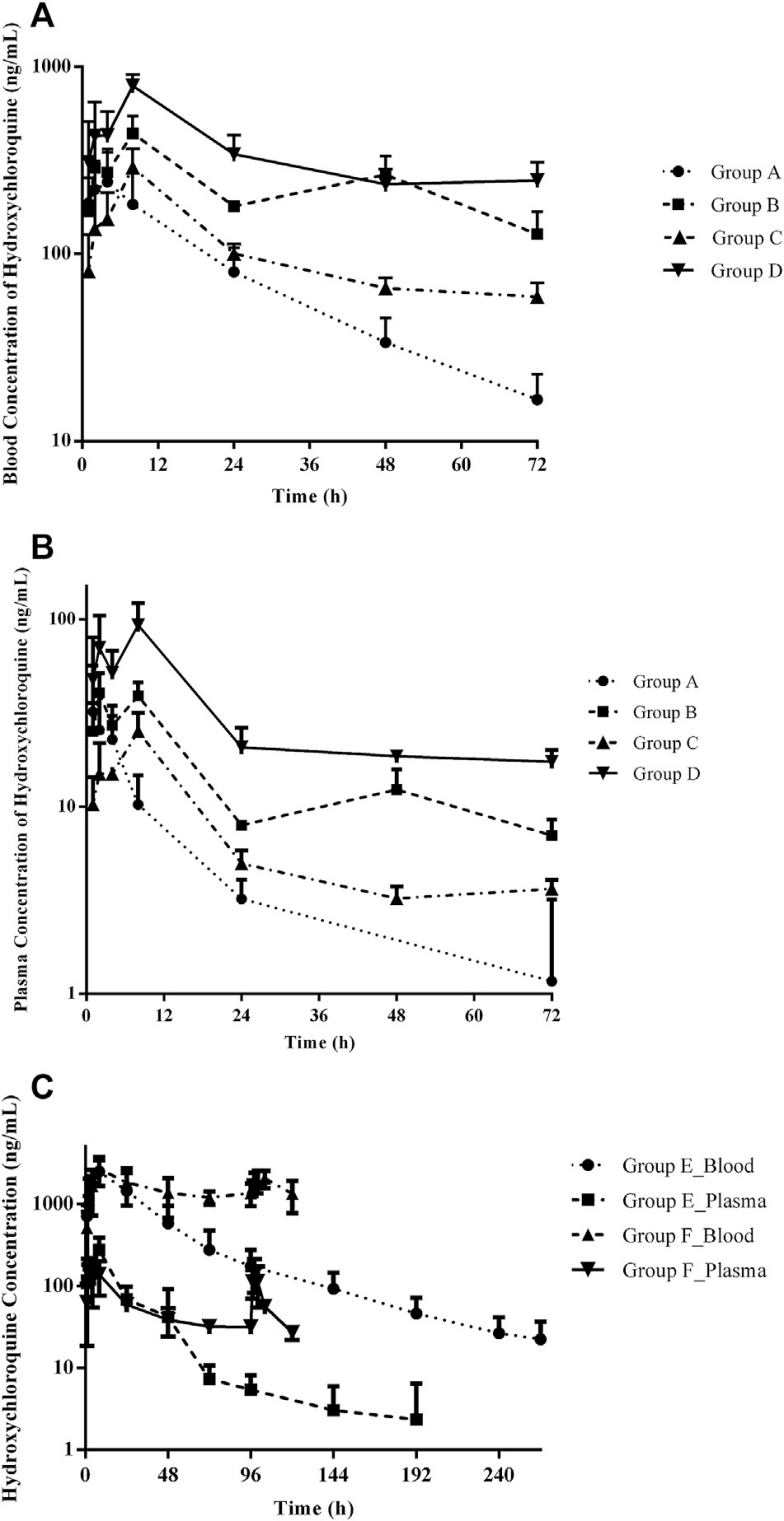
Summary of observed HCQ concentrations in the blood and plasma after different treatment regimens. Group A: 6 mg/kg twice (day 1) following 2 mg/kg twice daily (days 2–3); group B: 2 mg/kg twice (day 1) following 1 mg/kg twice daily (day 2–3); group C: 3 mg/kg twice (day 1) following 3 mg/kg twice daily (day 2–3); group D: 3 mg/kg (day 1); group E: 21 mg/kg twice (day 1); group F: 21 mg/kg twice (day 1) following 7 mg/kg twice daily (day 2–5). **(A)** Blood HCQ concentrations in groups A–D, **(B)** plasma HCQ concentrations in groups A–D, and **(C)** HCQ concentration in the blood and plasma, respectively, in groups E and F.

Initially, integrated models were established by considering four and five compartment models with and without an absorption lag phase or with and without peripheral compartment (model selection based on the likelihood ratio test) using measurements from the plasma, blood, and lung simultaneously. A model of the plasma, blood, and lung tissue was final developed by fitting a five-compartment model to represent the relationship between the plasma, blood concentrations, and lung tissue. The five-compartment model with first-order oral absorption (Ka, 1/h) and elimination (Ke, 1/h) was evaluation simultaneously to describe the time course of HCQ in the plasma (Figure 1). The parameters estimated in this structural PK model included Ka, volume of distribution for central compartment (Vc, L), two volumes of distribution for two peripheral compartments (Vb for blood and Vl for the lung tissue, respectively). Model parameters were assumed to follow a multivariate log-normal distribution, with stage 1 random error taken to be normally distributed with a proportional plus additive error variance.

Following Eqs (1)–(8) represent the PK model for HCQ in cynomolgus macaques:

$$\frac{d}{dt}A1=K_{a}\times A2+\;K_{bc}\times A3-K_{cb\max}\times \;\frac{A1}{A_{cb50}+A1}-K_{e}\times A1-K_{cl\max}\times \;\frac{A1}{A_{cl50}+A1}+\mathrm{Klc}\times A4-K_{cp}\times A1+K_{pc}\times A5$$ (1)

$$\frac{d}{dt}A2=-K_{a}\times A2$$ (2)

$$\frac{d}{dt}A3=K_{cb\max}\times \;\frac{A1}{A_{cb50}+A1}-\;K_{bc}\times A3$$ (3)

$$\frac{d}{dt}A4=K_{cl\max}\times \;\frac{A1}{A_{cl50}+A1}-K_{lc}\times A4$$ (4)

$$\frac{d}{dt}A5=K_{cp}\times A1-K_{pc}\times A5$$ (5)

$$Conc_{plasma}=\frac{A1}{V1}$$ (6)

$$Conc_{blood}=\frac{A3}{V3}+\frac{A1}{V1}$$ (7)

$$Conc_{lung}=\frac{A4}{V4}$$ (8)

where, A1, A2, A3, A4, and A5 denote the amount (mg) of concentration in the measured plasma compartment (central), absorption compartment, blood compartment, lung tissue compartment, and peripheral compartment, respectively. K_cbmax_ and K_clmax_ denote the maximum rate of transfer from the central compartment to the blood and lung, respectively, while A_cb50_ (mg) and A_cl50_ (mg) denote the amount of plasma compartments where the rate to blood is half-maximal of K_cbmax_ and K_clmax_, respectively. K_pc_ (1/h) and K_cp_ (1/h) denote the rate of transfer between the peripheral compartment and central compartment.

#### Model Evaluation

Model evaluation was performed using goodness-of-fit (GOF) plots with several diagnostic scatter plots and the comparison of observation and individual concentration time profiles, including (1) observed vs. population-predicted concentration (DV vs. PRED), (2) observed vs. individual-predicted concentration (DV vs. IPRED), (3) conditional standardized residuals vs. time (CSRES vs. TIME), and (4) conditional standardized residuals vs. population-predicted concentration (CSRES vs. PRED).

#### Pharmacokinetic Profile Simulation

The simulation of the pharmacokinetic profile following multiple HCQ doses was conducted using the typical parameters of the final model. The simulated HCQ concentration in the plasma, blood, and lung tissue was plotted over time.

#### Software and Platform Used

Population analysis was used to develop an overall pharmacokinetic model for describing the HCQ concentration sampled from each of the four components. Maximum likelihood estimates for model parameters were obtained through the application of the expectation maximization algorithm to the parametric, nonlinear mixed-effects maximum likelihood model, as proposed an developed by Schumitzky (Schumitzky, 1995) and Walker (Walker, 1996)and implemented in ADAPT (version 5, MLEM module) (D'Argenio et al., 2009). Model parameters were assumed to follow a multivariate log-normal distribution, with stage 1 random error taken to be normally distributed with a proportional plus additive error variance. Model simulation was performed by “SIM” module in ADAPT.

## Results

### Validation of HPLC-MS/MS

HCQ and DHCQ were spiked into blank plasma and various tissue samples, respectively, to determine the calibration curve. The linear responses for HCQ were observed over the corresponding concentration ranges of 5.00-1,000.00 ng/mL, 2.00-200.00 ng/mL, and 0.40-200.00 ng/mL in the blood, plasma, tissue, respectively. While the linear responses for DHCQ were observed over the corresponding concentration ranges of 2.50-500.00 ng/mL, 0.20-200.00 ng/mL, 0.40-200.00 ng/mL in the blood, plasma, tissue, respectively.

All the correlation coefficients were greater than 0.99, indicating a good linearity of the calibration curves. The lowest limit of quantification (LLOQ) of HCQ in blood, plasma, and tissue samples were 5.00 ng/mL, 2.00 ng/mL and 0.40 ng/mL, respectively. While the lowest limit of quantification (LLOQ) of DHCQ in blood, plasma, and tissue samples were 2.50 ng/mL, 0.20 ng/mL, and 0.40 ng/mL, respectively.

### HCQ Pharmacokinetics

The developed and validated LC–MS/MS method was successfully applied to the determination of HCQ pharmacokinetics and tissue distribution after intragastric administration of HCQ in monkeys following the dose regimen described in “Section 2.5.” The blood and plasma concentrations vs. time profiles for the HCQ are shown in Figure 1. The maximum concentration (C_max_) of HCQ was 292.33 ± 114.66 ng/mL in blood and 36.90 ± 22.52 ng/mL in plasma. The value of area under curve (AUC_0–∞_) were determined as 5,978.94 ± 1981.30 and 363.31 ± 195.38 h*ng/mL for blood and plasma, respectively. HCQ were detected in the blood and plasma up to 72 h, which was the last time point monitored. The percentage extrapolation of the AUC from the last measured time point to infinity was less than 10% and 20% in the blood and plasma, respectively.

### Biodistribution

Concentrations of HCQ and DHCQ were detected in all studied tissues (Tables 1–3). In addition, our data showed that all the tissues to blood concentration ratio (Kp__tissue/blood_) were ≥1, indicating accumulation of HCQ in tissues. The HCQ Kp__tissue/blood_ ratio for the various tissues was observed in the descending order of thymus > kidney > spleen > liver > heart > subcutaneous fat > brain > lung in group E, whereas the Kp__tissue/blood_ ratio was different in group F with the descending order of liver > thymus > spleen > kidney > lung > heart > subcutaneous fat > brain. But the last sampling time of group E was 264 h, and the corresponding plasma concentration was below the limit of quantification, resulting the Kp__tissue/plasma_ was only available in group F. Similar to the Kp__tissue/blood_ ratio, the descending order of the Kp__tissue/plasma_ ratio in group F was liver > spleen > kidney > lung > heart > subcutaneous fat > brain. The Kp__tissue/plasma_ ratio and Kp__tissue/blood_ ratio for the lung was 267.38 ± 42.93 and 5.55 ± 0.46, respectively.

**TABLE 1:** Tissues concentration (ng/g) and Kp__tissue/blood_ ratio of HCQ (mean ± SD).

	**Thymus (ng/g)**	**Kidney (ng/g)**	**Spleen (ng/g)**	**Liver (ng/g)**	**Heart (ng/g)**	**Subcutaneous Fat (ng/g)**	**Brain (ng/g)**	**Lung (ng/g)**	**BALF (ng/mL)**
Group E: tissue concentration	481.33 ± 242.24	79.70 ± 15.31	82.30 ± 34.37	78.83 ± 28.77	65.23 ± 21.37	35.22 ± 35.22	33.10 ± 11.46	20.77 ± 8.57	26.83 ± 11.19
Group E: Kp ratio	223.25 ± 23.79	43.33 ± 22.42	39.91 ± 13.85	38.63 ± 11.25	32.16 ± 7.80	20.68 ± 25.51	16.40 ± 5.19	9.98 ± 2.09	1.33 ± 0.63
Group F: tissue concentration	1,375.00 ± 219.20	1,115.00 ± 7.07	1,610.00 ± 1,032.38	3,025.00 ± 1,435.43	579.50 ± 120.92	75.55 ± 4.74	59.85 ± 11.81	738.50 ± 256.68	168.50 ± 40.31
Group F: Kp ratio	11.54 ± 6.52	9.07 ± 3.90	11.29 ± 2.83	22.09 ± 1.22	4.91 ± 2.98	0.61 ± 0.22	0.47 ± 0.11	5.55 ± 0.46	0.13 ± 0.03

**TABLE 2:** Tissues concentration (ng/g) and Kp__tissue/blood_ ratio of DHCQ (mean ± SD).

	**Liver (ng/g)**	**Heart (ng/g)**	**Spleen (ng/g)**	**Kidney (ng/g)**	**Thymus (ng/g)**	**Lung (ng/g)**	**Subcutaneous Fat (ng/g)**	**Brain (ng/g)**	**BALF (ng/mL)**
Group E: tissue concentration	1,541.00 ± 847.01	304.00 ± 58.39	214.33 ± 61.16	238.33 ± 72.95	186.00 ± 30.51	41.20 ± 9.80	33.13 ± 11.79	7.06 ± 2.48	30.63 ± 17.05
Group E: Kp ratio	114.79 ± 67.47	22.48 ± 3.38	16.08 ± 5.46	17.86 ± 6.29	13.79 ± 2.00	3.10 ± 0.94	2.43 ± 0.70	0.54 ± 0.23	0.23 ± 0.13
Group F: tissue concentration	3,315.00 ± 657.61	747.00 ± 203.65	1715.00 ± 968.74	1,126.00 ± 189.50	560.00 ± 138.59	307.00 ± 96.17	68.55 ± 13.08	21.95 ± 9.83	71.55 ± 18.60
Group F: Kp ratio	18.57 ± 4.11	4.16 ± 1.04	9.65 ± 5.64	6.28 ± 0.91	3.12 ± 0.70	1.72 ± 0.58	0.38 ± 0.06	0.12 ± 0.06	0.04 ± 0.01

**TABLE 3:** Tissues concentration (ng/g) and Kp__tissue/plasma_ ratio of HCQ (mean ± SD).

	**Liver (ng/g)**	**Spleen (ng/g)**	**Thymus (ng/g)**	**Kidney (ng/g)**	**Lung (ng/g)**	**Heart (ng/g)**	**Subcutaneous Fat (ng/g)**	**Brain (ng/g)**	**BALF (ng/mL)**
Group F: tissue concentration	3,025.00 ± 1,435.43	1,610.00 ± 1,032.38	1,375.00 ± 219.20	1,115.00 ± 7.07	738.50 ± 256.68	579.50 ± 120.92	75.55 ± 4.74	59.85 ± 11.81	168.50 ± 40.31
Group F: Kp ratio	1,081.38 ± 319.70	565.87 ± 270.69	522.94 ± 181.19	417.91 ± 82.99	267.38 ± 42.93	221.42 ± 87.05	28.13 ± 3.67	21.99 ± 0.11	6.17 ± 0.30

### Model Development and Evaluation

**FIGURE 2:**
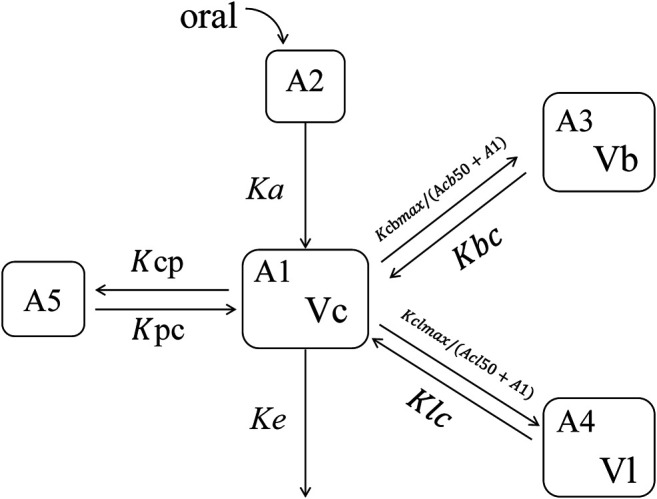
Schematic representation of the pharmacokinetic model used to describe HCQ concentration–time profiles after oral administration. A, amounts; K_a_, the rate of constant of absorption; K_e_, the rate of elimination; V_c_, central volume of distribution; V_b_, red blood cell volume of distribution; VL, lung volume of distribution; K_cbmax_, the maximum drug transport rate from the central compartment to red blood cells; K_clmax_, the maximum drug transport rate from the central compartment to the lung tissue; A_cb50_, the HCQ amount resulting in 50% of maximum transport rate from the central compartment to red blood cells; K_bc_, the rate of transport from red blood cells to the central compartment; A_cb50_, the HCQ amount resulting in 50% of maximum transport rate from the central compartment to the lung tissue; K_lc_, the rate of transport from the lung tissue to the central compartment; K_pc_ and K_cp_, the rate constant between the peripheral compartment and central compartment.

A five-compartment model with first-order oral absorption and elimination was selected as the base model for HCQ concentration in the plasma, blood, and lung tissue (Figure 2). The absorption rate Ka of plasma HCQ was estimated to be 0.592 h^−1^, and Ke was estimated to be 0.236 h^−1^. The volume of distribution in the central (Vc/F) and other two peripheral compartments (Vb/F and Vl/F) were 114, 2.68 and 5.55 L, respectively. Estimates of A_cb50_, A_cl50_, K_bc_, K_lc_, K_cbmax_, K_clmax_, K_cp_, and K_pc_ between the central compartment and different peripheral compartments were 7.05 mg, 0.498 mg, 0.718 h^−1^, 0.159 h^−1^, 2.48 h^−1^, 1.92 h^−1^, 0.600 h^−1^, and 0.514 h^−1^, respectively. The interindividual variability (IIV) was estimated for Ka, Ke, Vc/F, Vb/F, Vl/F, A_cb50_, A_cl50_, K_bc_, K_lc_, K_cbmax_, K_clmax_, K_cp_, and K_pc_. The IIV for PK parameters were expressed as CV% and ranged from 29 to 88.7%, except for IIV of K_cl_ (160%). The final model parameter estimates are summarized in Table 4.

**TABLE 4:** Model parameter estimation for the final pharmacokinetic model of HCQ in cynomolgus macaques.

**Parameter**	**Description**	**Mean** ^#^	**IIV CV%** *
K_e_ (1/h)	Elimination rate constant from the central compartment (plasma)	0.236	54.3
V_c_/F (L)	Apparent volume of the central compartment (plasma)	114	56.3
K_a_ (1/h)	Absorption rate constant	0.592	29
A_cb50_ (mg)	Amount of the plasma compartment where the rate to blood is half-maximal	7.05	120
K_bc_ (1/h)	Rate constant from the blood compartment to plasma	0.718	52.6
V_b_/F (L)	Blood compartment volume of distribution	2.68	125
V_L_/F (L)	Lung compartment volume of distribution	5.55	86
K_lc_ (1/h)	Rate constant from the lung to central compartment	0.159	94.5
A_cl50_ (mg)	Amount of central compartment where the rate to lung is half-maximal	0.498	222
*K*_cbmax_	Maximum rate constant from the central compartment to blood	2.48	131
K_clmax_	Maximum rate constant from the central compartment to lung	1.92	78.9
K_pc_ (1/h)	Rate constant from the peripheral compartment to central compartment	0.514	88.7
K_cp_ (1/h)	Rate constant from central compartment to peripheral compartment	0.600	122
SD_pl_ (ng/mL)	Standard deviation of the additive residual error for plasma	0.100E−04	Fixed
PD_pl_	Proportional residual error for plasma	0.286	
SD_bl_ (ng/mL)	Standard deviation of the additive residual error for blood	0.100E−04	Fixed
PD_bl_	Proportional residual error for blood	0.118	
SD_lu_ (ng/g)	Standard deviation of the additive residual error for lung tissue	0.100E−04	Fixed
PD_lu_	Proportional residual error for lung tissue	0.514E−01	

* IIV: Interindividual variability; CV: coefficient of variation.

^#^ Due to limited sample size, the RSD% cannot be estimated.

**FIGURE 3:**
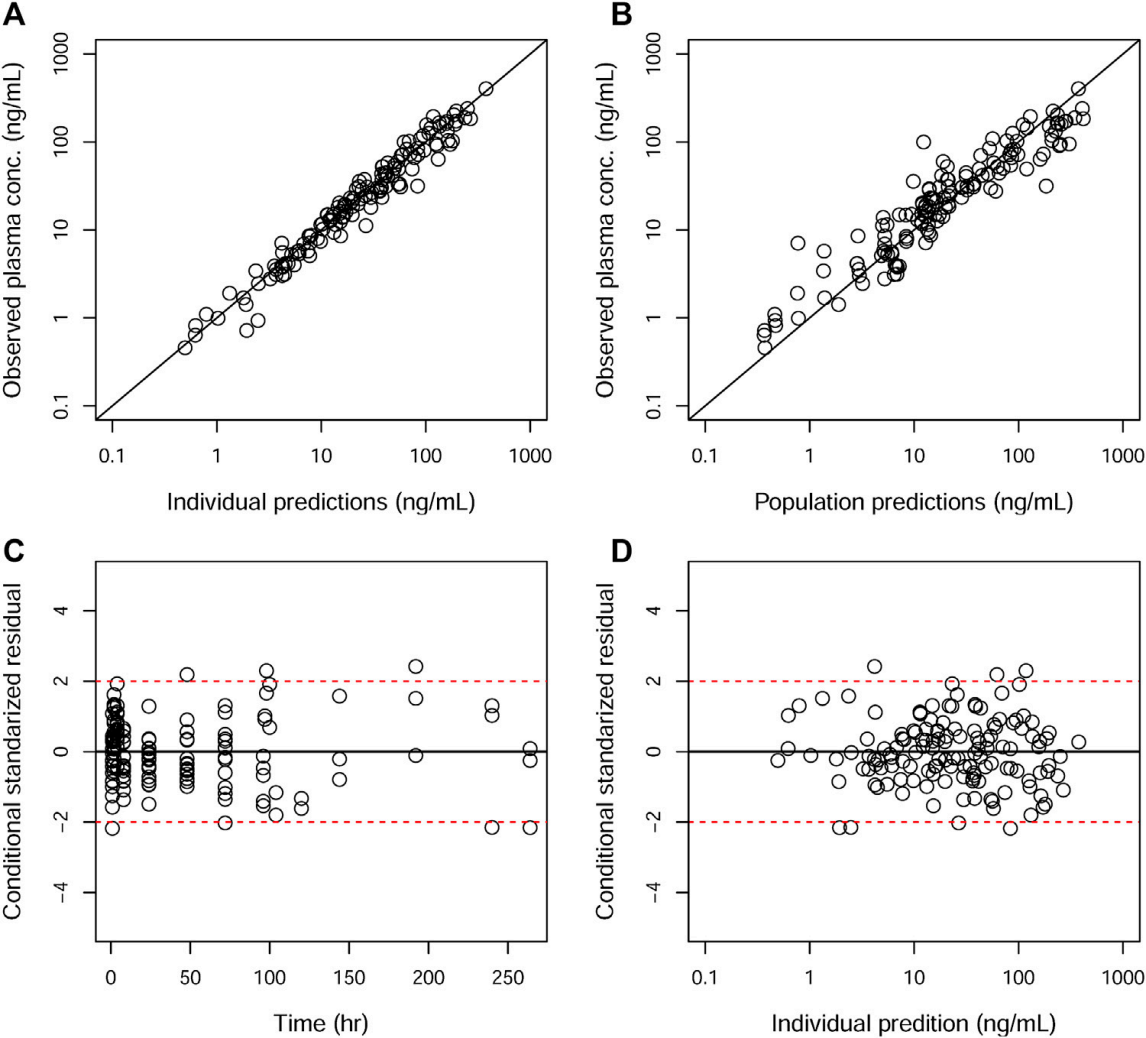
Goodness-of-fit plots of the plasma for the final model. The hollow circles represent the observed data from individual monkeys. The black diagonal **(A and B)** and horizontal lines **(C and D)** are the lines of identity and zero lines, respectively. The dashed lines in the bottom panel are the conditional standardized residual equal ±2.

**FIGURE 4:**
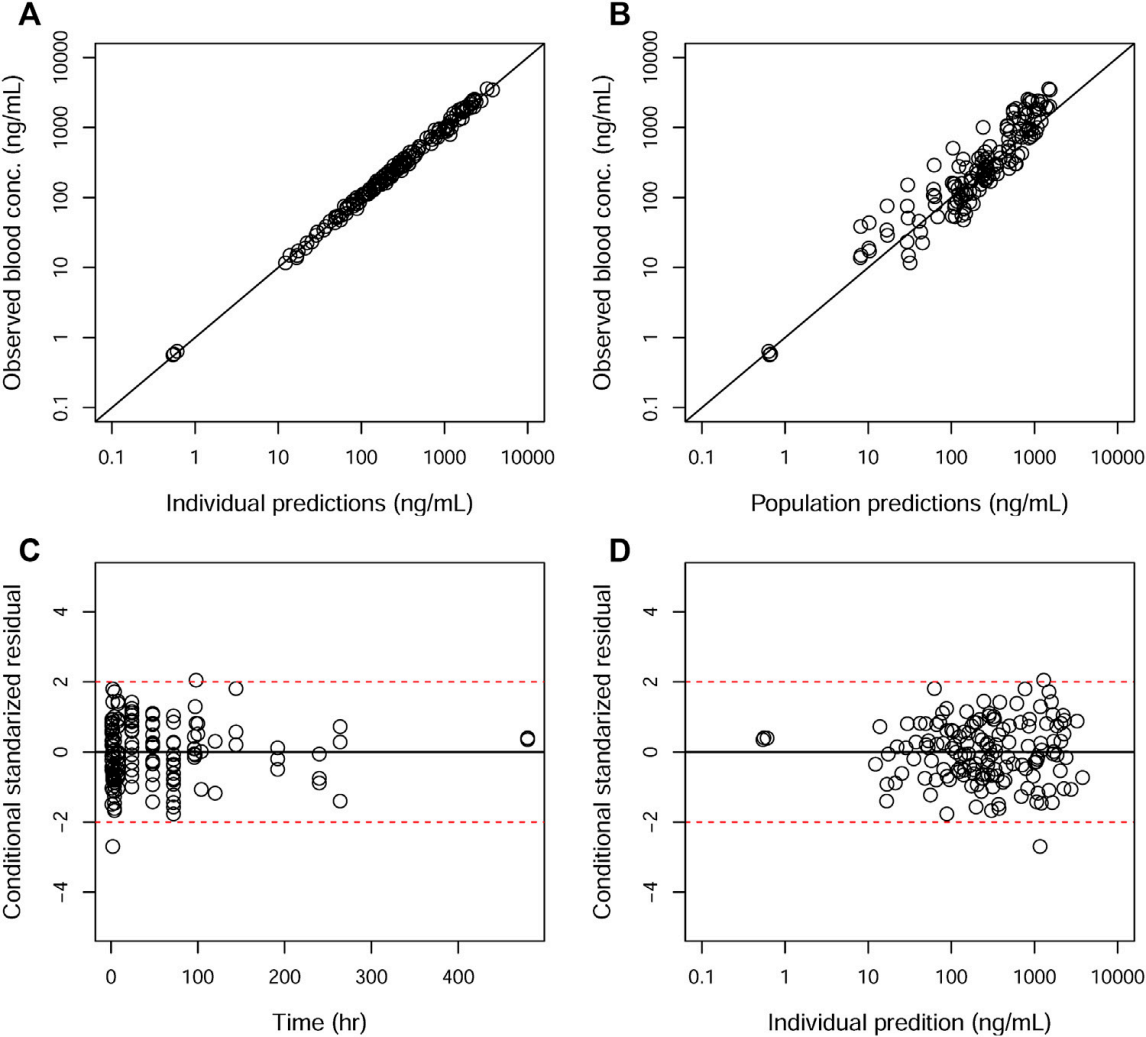
Goodness-of-fit plots of the blood for the final model. The hollow circles represent the observed data from individual monkeys. The black diagonal **(A and B)** and horizontal lines **(C and D)** are the lines of identity and zero lines, respectively. The dashed lines in the bottom panel are the conditional standardized residual equal ±2.

**FIGURE 5:**
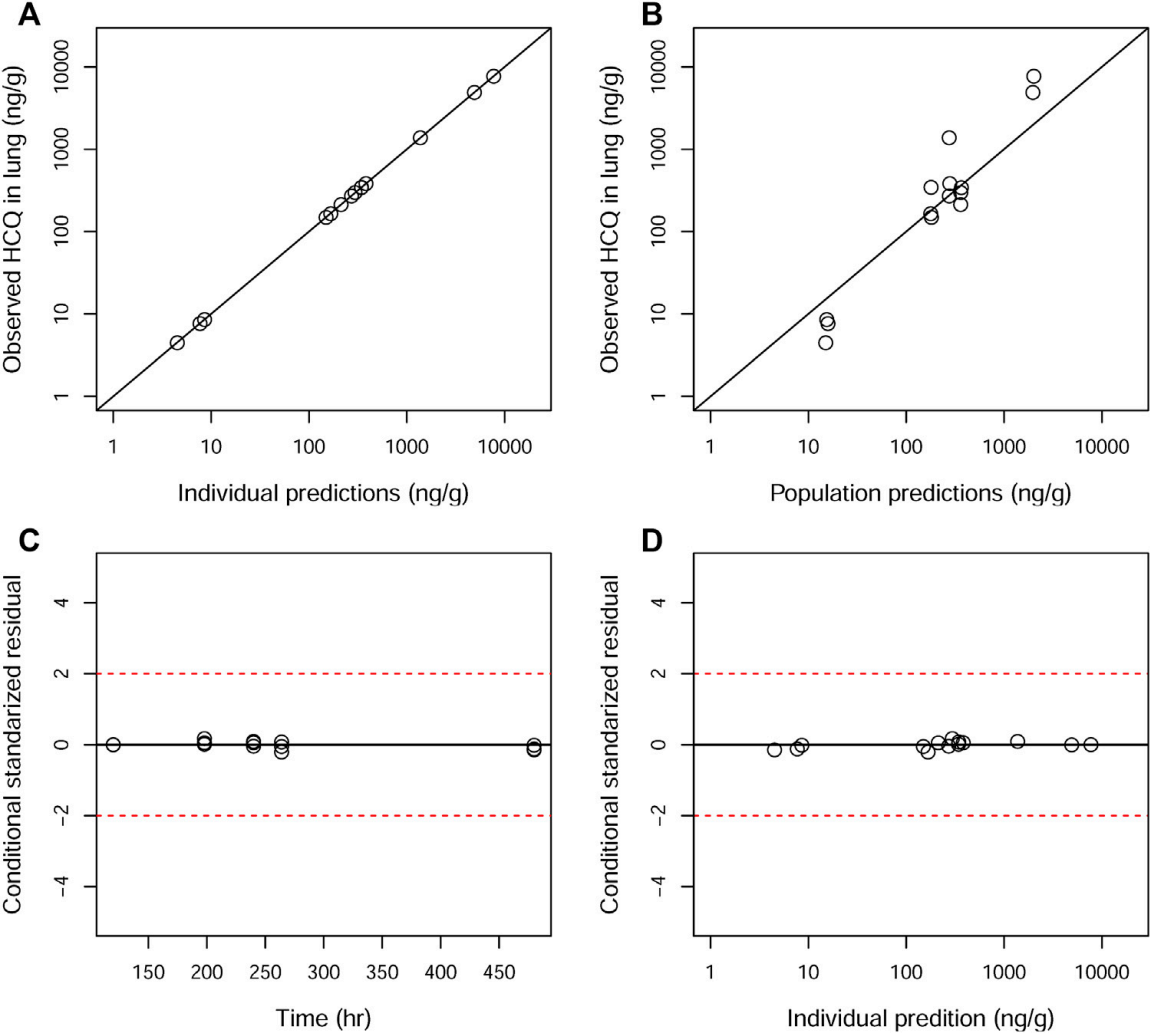
Goodness-of-fit plots of the lung for the final model. The hollow circles represent the observed data from individual monkeys. The black diagonal **(A and B)** and horizontal lines **(C and D)** are the lines of identity and zero lines, respectively. The dashed lines in the bottom panel are the conditional standardized residual equal ±2.

**FIGURE 6:**
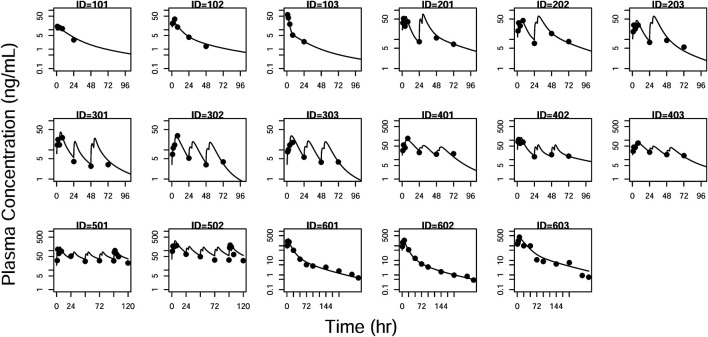
Individual prediction plots for plasma concentration. The symbols are the observed data. The lines are the individual fitting lines from final PK model.

**FIGURE 7:**
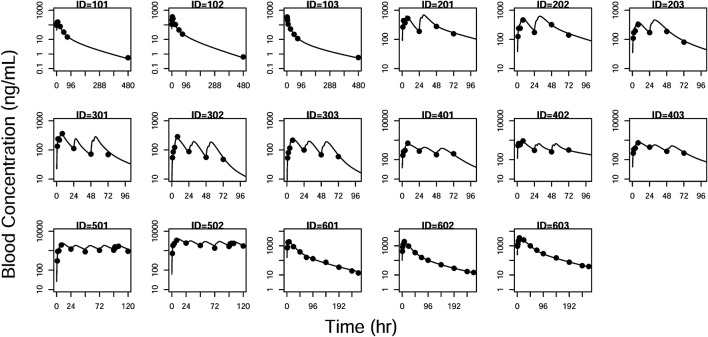
Individual prediction plots for blood concentration. The symbols are the observed data. The lines are the individual fitting lines from final PK model.

**FIGURE 8:**
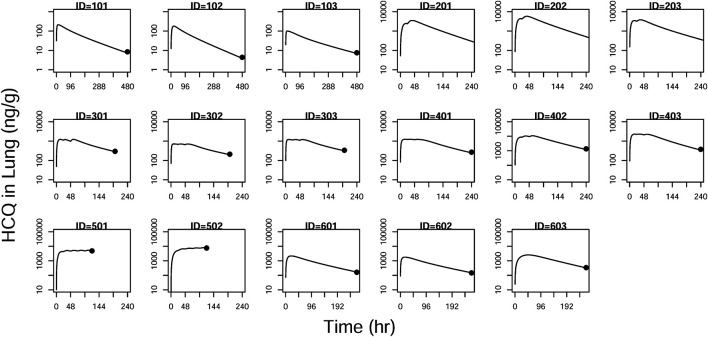
Individual prediction plots for concentration in the lung. The symbols are the observed data. The lines are the individual fitting lines from final PK model.

GOF plots for the final model were generated, as depicted in Figures 3–5. The population- and individual-predicted plasma, blood, and lung HCQ concentrations vs. observed concentrations showed no major bias. The conditional standardized residuals (CSRES) vs. time or vs. individual-predicted concentrations showed that most of CSRES were within the range of (−2, 2). These model diagnostics suggested that the final population PK model of the monkey to adequately describes the HCQ plasma, blood, and lung data. Figures 6–8 display the individual prediction GOF of HCQ concentration in the plasma, blood, and lung tissue for the final model.

### Model Simulation

**FIGURE 9:**
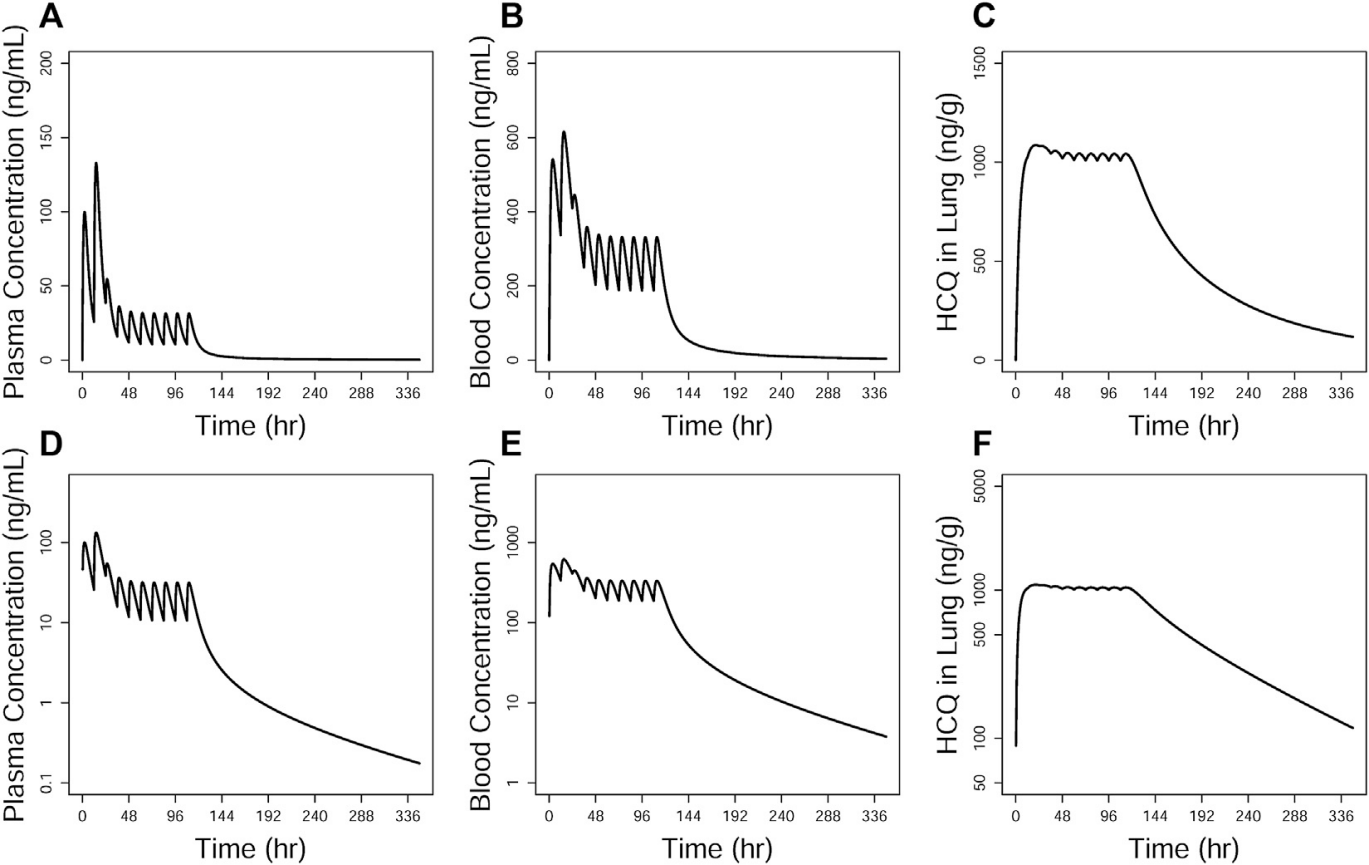
Simulations of HCQ pharmacokinetic profiles in the plasma **(A and D)**, blood **(B and E)**, and lung tissue **(C and F)**, respectively. The upper row is constant coordinate plots and the lower row semi-log ones.

One dosing regimen of HCQ was simulated using the final population PK model. The simulated HCQ mean concentration in plasma, blood, and lung tissue is shown in Figure 9. And there were also simultaneously simulated the ratios of the lung tissue to plasma in different time points after dosing, as shown in Table 5.

**TABLE 5:** Simulated HCQ concentration data in the plasma, blood and lung, and the ratio of lung/plasma based on the final population PK model.

**TAD (hr)**	**Plasma (ng/mL)**	**Blood (ng/mL)**	**Lung (ng/g)**	**Ratio of lung/plasma**
12	10.63	187.29	1,008.54	94.85
24	4.26	85.40	865.68	203.00
48	1.80	37.68	629.94	349.33
72	1.10	23.19	482.23	440.31
96	0.76	16.11	380.55	503.46

## Discussion

SARS-CoV-2 can attack cells expressing angiotensin I-converting enzyme 2 (ACE2), leading to serious infections of the lungs, the gastrointestinal tract, and other tissues (Hamming et al., 2004). Moreover, antiviral drugs are needed to penetrate the multiple sites where SARS-CoV-2 infection occurs with sufficient concentrations to inhibit viral replication. Global research efforts are focused on screening the activity of existing compounds *in vitro* to identify candidates to repurpose for SARS-CoV-2. The antimalarial HCQ has demonstrated antiviral activity against SARS–CoV-2 *in vitro* and in several clinical studies with small sample size. However, the efficacy of HCQ in the treatment of COVID-19 was quite controversial in clinical studies. It appeared that the free plasma concentration of HCQ was lower than extracellular EC_50_ *in vitro* (submitted data). However, it is quite complicated to extrapolate efficacy from *in vitro* to humans just based on this simple comparison, as pointed out by Jansson-Löfmark et al. (2020). Moreover, HCQ has specific affinity with pigmented tissues, lysosomes, and golgi (Hoekenga, 1954; Fox, 1993; Schroeder and Gerber, 2014). We later measured intracellular EC_50_, which could be compared with lung tissue concentration, and there are still many challenges, such as possible differences in mechanism, active metabolites (unpublished data). Although we knew more about its cellular kinetics and its distribution in monkeys, the recommendation of appropriate dosage to treat COVID-19 still needs to consider safety and efficacy margin to allow possible difference between *in vivo* and *in vitro*.

Considering that it is impossible to carry out biodistribution research in clinical studies, the additional pharmacokinetics experiments in animals are necessary to understand its exposure in tissues. It is especially helpful when the PBPK modeling strategy was recognized to be supportive but need more validated data, such as lung tissue or lung lining fluid concentrations (Rowland Yeo et al., 2020b; Maharaj et al., 2020). It has been reported the elimination rate of the drug was rapid in mice and its tissue distribution kinetics data are very different from those of human tissue distribution (Chhonker et al., 2018). Thus, it is necessary to perform the distribution kinetic study in monkeys in order to understand the distribution characteristics of HCQ to predict human pharmacokinetics with more confidence. These are essential information for further studies of the pharmacokinetics/pharmacodynamics relationship of HCQ. Higher concentrations in different tissues indicated that further studies are required to investigate the relationship between drug concentration and efficacy, both therapeutic and toxicity, after therapeutic regimens. There is little information regarding tissue distribution of HCQ in monkeys, even less information about metabolite concentrations accumulating in tissues. To evaluate the therapeutic effects on COVID-19 and drug toxicity, it is critical that assays be developed that are sensitive enough to quantitate drug concentrations in tissues, especially the lungs. Moreover, the relationship between metabolite concentration and efficacy or toxicity is unclear. Developing an enhanced understanding of the PKs of HCQ and its metabolites in animal models (including their concentrations in tissue and corresponding kinetics), and their interaction with other therapies is crucial in supporting the clinical efficacy and safety, characterizing and further optimizing combination therapies involving HCQ.

The pharmacokinetics data obtained from different dosing regimens could be used simultaneously to perform population pharmacokinetics model and evaluate the pharmacokinetics characteristic of HCQ. Since the HCQ concentration in the lung tissue is higher than that in the plasma or blood, we could confirm that it might be effective in the lung. In addition, there has been simulated that lung pH reduction from 6.7 to 6 in COVID-19 patients could cause 4.0-fold increases in lung exposure of HCQ, indicating that higher concentrations of HCQ in patients’ lung tissue with COVID-19 were sustained for a longer period after administration (Rowland Yeo et al., 2020a). Many other weakly basic drugs have also been demonstrated to accumulate in lysosomes (Ohkuma and Poole, 1981; Duvvuri and Krise, 2005). However, there are few data about the population pharmacokinetics model to describe the characteristic of these drugs. Furthermore, with the simulation results of multidose regimen, the accumulated concentration in the lung tissue was more than that in the blood or plasma, and the rate of HCQ concentration decrease was faster in the circulation system than that in the lung tissue, indicating that there was a dynamic change course as shown by the ratio of the blood or plasma HCQ concentration to the lung tissue HCQ concentration, which could be used to provide information for HCQ clinical treatment.

Finally, as stereoisomeric drug, HCQ is administered as racemates, whereas enantiomeric differences may be existing in pharmacokinetics (Iredale et al., 1993; Tett, 1993). Therefore, in order to better understand the pharmacokinetics of enantiomers of HCQ on efficacy and safety, it is necessary to design the studies with two isomeric forms of HCQ.

## Conclusion

In summary, a population pharmacokinetics model for HCQ in cynomolgus macaques was developed and constructed using data from monkeys. The derived population PK model should be able to evaluate the dynamic process of HCQ in the plasma, blood, and lung tissue concentration to support the clinical applications.

## Data Availability Statement

The raw data supporting the conclusions of this article will be made available by the authors, without undue reservation.

## Ethics Statement

The animal study was reviewed and approved by Institutional Animal Care and Use Committee(IACUC) Pharmaron Beijing Co., Ltd, Beijing, China.

## Author Contributions

DL conceived and designed the study. HL and CS directed this whole study. KW developed and optimized the hydroxychloroquine population PK model. QL and GB designed the dose regimen of hydroxychloroquine and analyzed the experiment data. XG and ST performed population PK model analysis. XT, MX, and JW carried out the animal experiments. GC, ML, BW, and HJ conducted the bioanalysis of hydroxychloroquine. QL, GB, and DL prepared this manuscript.

## Funding

This work was supported by the Bill & Melinda Gates Foundation (INV-015694), the Ministry of Science and Technology of the People’s Republic of China (Grant No. 2020YFC0844500), and the National Natural Science Foundation of China (Project Nos. 81672133 and 81874010).

## Conflict of Interest

Authors GC, ML, BW, and HJ were employed by the company HR-Biopharm Technology Co., Ltd. Authors XT, MX, and JW were employed by the company Pharmaron Beijing Co., Ltd. Author KW was employed by the company Shanghai Qiangshi Information Technology Co., Ltd.

The remaining authors declare that the research was conducted in the absence of any commercial or financial relationships that could be construed as a potential conflict of interest.
